# Supplementary material for: MetaRibo-Seq measures translation in microbiomes
Source: Nat Commun. 2020 Jun 29;11:3268. doi: 10.1038/s41467-020-17081-z (PMC7324362; doi:10.1038/s41467-020-17081-z)
Supplement: Supplementary file 10 — Supplementary Data 7 [file 41467_2020_17081_MOESM10_ESM.zip › File2/Confidence_High_Taxonomy/196955_out.krona.html]

Javascript must be enabled to view this page.

members
magnitude
magnitudeUnassigned
count
unassigned
taxon
rank

196955\_out

2

2
2
superkingdom

phylum
2
1239

1
91061
class

186826
1
order

1300
1
family

1301
1
genus

1
1318

SRS104476\_contig\_number\_contig-100\_22866.54801
species

186801
1
class

order
186802
1

family
1
31979

1
1485
genus


SRS147977\_contig\_number\_contig-100\_22713.22714
species
1
1650661
